# Effort and performance in a cooperative activity are boosted by perception of a partner’s effort

**DOI:** 10.1038/s41598-018-34096-1

**Published:** 2018-10-24

**Authors:** Matthew Chennells, John Michael

**Affiliations:** 1 Philosophy Department, University of Warwick, Coventry, UK and Department of Cognitive Science, Central European University, Budapest, Hungary; 2 Philosophy Department, University of Warwick, Coventry, UK and Department of Cognitive Science, Central European University, Budapest, Hungary

## Abstract

In everyday life, people must often determine how much time and effort to allocate to cooperative activities. In the current study, we tested the hypothesis that the perception of others’ effort investment in a cooperative activity may elicit a sense of commitment, leading people to allocate more time and effort to the activity themselves. We developed an effortful task in which participants were required to move an increasingly difficult bar slider on a screen while simultaneously reacting to the appearance of virtual coins and earn points to share between themselves and their partner. This design allowed us to operationalize commitment in terms of participants’ investment of time and effort. Crucially, the cooperative activity could only be performed after a partner had completed a complementary activity which we manipulated to be either easy (Low Effort condition) or difficult (High Effort condition). Our results revealed participants invested more effort, persisted longer and performed better in the High Effort condition, i.e. when they perceived their partner to have invested more effort. These results support the hypothesis that the perception of a partner’s effort boosts one’s own sense of commitment to a cooperative activity, and consequently also one’s willingness to invest time and effort.

## Introduction

Cooperation can be observed in a wide range of species, from bees swarming^1^ to rooks jointly pulling a baited platform into their cage^2^, and chimpanzees hunting for monkeys^3^. As humans, however, we are unique in our capacity to cooperate flexibly across a wide variety of contexts, despite tremendous individual diversity, complex environments and uncertainty about the future^4–6^. While it may be unsurprising that people are willing to contribute to cooperative activities when the benefits of doing so outweigh the costs, it is more difficult to explain why they do so in situations where it is unclear that contributing is in their best interest.

In recent decades, a great deal of research in evolutionary theory, experimental economics and psychology has been devoted to investigating this issue^4,7–10^. This research has led to substantial progress in identifying ultimate (i.e. evolutionary) mechanisms that are likely to have supported the evolution of cooperation in humans, such as kin selection^11,12^, direct^13^ and indirect^14^ reciprocity, interdependence^15^, and cultural group selection;^16^ and in identifying proximate psychological mechanisms underpinning cooperation, such as other-regarding preferences for equality^17^, an aversion to guilt^18^, and an aversion to disappointing others’ expectations^19^.

Most of this research has focused on situations in which the costs and benefits that are at stake are monetary. This can make it possible to quantify and compare the value of cooperation for people under a wide range of circumstances, although it is important to be cautious insofar as the monetary scale is not guaranteed to be proportional to the perceived value. In everyday life, however, people often must make decisions about contributing less easily quantifiable resources, in particular effort, in cooperative activities. In these cases, it may be more difficult for them to assess the level of others’ contributions, to compare others’ contributions with their own, and to determine the amount that is appropriate for them to contribute. To attain a fuller understanding of the psychological underpinnings of human cooperation, it is important to investigate people’s decisions and actions in these types of situation.

Over the past few years, researchers have begun to turn their attention to this challenge. Braun, Ortega and Wolpert (2009, 2011)^20,21^, for example, implemented a battery of social dilemmas with structures deriving from behavioural economics but in which the resource at stake is physical effort–e.g. participants can cooperate to minimize global effort in a prisoners’ dilemma, or attempt to minimize their own individual effort at the expense of their partner. The main finding is that cooperation rates are much lower than typically observed in standard versions of the prisoners’ dilemma. In a similar vein, Lockwood, Husain and Apps (2017)^22^ found that participants were less willing to exert physical effort to generate monetary rewards for others than when the reward was for themselves. These recent findings raise the important question: Under what circumstances *are* people willing to invest a high degree of effort in cooperative activities?

## The Current Research

We hypothesized that one important factor may be the perception of a partner’s effort. Specifically, the perception of a partner’s effort may elicit a sense of commitment to a cooperative activity, thereby boosting the willingness to reciprocate by contributing more time and effort as well^23^. To test this, we implemented a cooperative task requiring two players to contribute distinct, complementary forms of effort: one player (the role of which, unbeknownst to participants, was played by a virtual agent) must perform a cognitive task in order to ‘unlock’ each new round of a game in which the other player (the participant) must invest physical effort in order to attain points that would be converted at the end of the experiment into monetary rewards to be shared equally by both players.

Specifically, the participants’ task was to repeatedly press a spacebar to advance a cursor along a progress bar, thereby causing virtual coins to appear at unpredictable intervals. In addition, each time a virtual coin appeared, their task was to press the ‘*s*’ key within one second to retrieve the coin. Over the course of each round, it became increasingly difficult to advance the cursor, i.e. participants had to press the space bar increasingly quickly to make progress. As a result, the effort cost increased progressively over each round, creating an increasingly strong temptation to disengage from the task – which participants could freely do whenever they chose to – by pressing a button that would end the current round and initiate a new round. Participants perceived the likelihood of coin appearance to be evenly distributed across the line; they therefore make slower progress in progressing along the line because of the increasing effort cost required to advance the cursor. But since the coins increased in value over the course of each round, the optimal strategy with respect to maximizing the joint points total, and hence the partner’s monetary reward, was to continue each round until the maximum number of coins had been retrieved. This enabled us to operationalize participants’ commitment to the cooperative task by analysing their persistence in obtaining as many points as possible.

By modulating the apparent difficulty of the cognitive task performed by their partner at the beginning of each round, we were able to manipulate the degree of effort which participants perceived their partner to be investing. We hypothesized that on rounds on which participants perceived their partner investing a high degree of effort (High Effort Condition: HE), they would be more committed to the task than on rounds when they perceived their partner investing a low degree of effort (Low Effort Condition: LE). To ensure that they could not directly compare their own and their partner’s effort level, we implemented a design in which their task is different from that of their partner. If it were possible to directly compare their effort level with that of their partner, we were concerned that participants would use their partner’s effort contribution as an anchor for their own.

Our experimental design allowed us to operationalize participants’ commitment to the cooperative activity in four ways. First, through performance, i.e. the amount of *points earned* within a round. We predicted that higher perceived partner effort in HE rounds would lead to greater commitment by participants to the joint goal of maximising the number of coins “caught” and, consequently, points earned. Second, we expected participants to persist for longer before choosing to end the round in HE relative to LE rounds, resulting in more time spent on the task and longer *round length*. Third, we predicted that more committed participants in the HE condition would choose to exert more effort to move the yellow line further along before exiting, manifesting in a larger number of *key presses* within HE versus LE rounds. Fourth, we reasoned that high commitment in the HE condition should lead participants to “hang on” until a coin appears rather than exiting the round even after they have lost interest in the round. If so, then we should expect participants in the HE condition to tend to exit rounds *right after appearances of coins*. This means that in the HE condition we should expect to see fewer key presses after the appearance of the final coin (*post-coin key presses*).

Our experimental design also allowed us to explore the cognitive mechanisms underpinning the behavioural effects we predicted. Specifically, we were interested in whether a sense of commitment also acts as a top-down executive control mechanism, working to maintain representation of the value of the task to the partner and/or the cost invested by the partner in working memory^24^. In addition to pressing the key to move the yellow line, participants were tasked with reacting to the appearance of coins (with point values). When coins appeared, participants had one second to recover the coin before it disappeared. Differences in executive control may thus be revealed by differences in *reaction times* (RTs). We did not make any prediction about the direction of the effect for this measure. This is because increased motivation to recover coins may lead to lower RTs in the high effort condition, but increased attention to the key-pressing task might also distract participants from the additional task of recovering coins as they appear. In addition, we measured the *speed* of participants’ keypresses–i.e. the frequency with which participants pressed the key to move the yellow line over the duration of the round. We predicted that heightened executive control would translate into greater consistency in effort application, with more constant attention paid to coin appearance and more constant key-pressing. We thus expect to find less variability in both reaction times and speed in HE rounds compared to LE rounds.

## Results

For the analysis, we excluded participants who did not complete at least 8 rounds (minimum 4 in each condition) in which they exited the round. This was an *a priori* decision made to ensure we would have enough data to compute means for each condition. We also limit our sample to only those rounds in which participants exited the round (92% of all rounds). Our analysis thus excludes rounds which participants were performing when the maximum duration of the experiment was reached (30 minutes) and the experiment automatically ended (6% of all rounds), and those rounds in which participants ended the round by reaching the end of the line (2% of all rounds). These rounds are dropped as they contain an upper bound; there is no counterfactual as to how long participants might have persisted had they been given the opportunity. Our sample of 27 participants contains 396 rounds, an average of 14.67 rounds per person. Approximately half (51%) the rounds faced are classified as LE condition and the rest (49%) as HE condition.

To test the data for normality and homogeneity of variance we conducted Shapiro-Wilk tests for all our measured variables (A-D), from which the following p-values obtained: (A) Mean number of coin appearances per round: HE condition, *p* = 0.0005, and LE condition, *p* = 0.0193; (B) Mean number of key presses per round: HE condition, *p* = 0.0599, and LE condition, *p* = 0.3219; (C) Mean round length, in seconds: HE condition, *p* = 0.0003, and LE condition, *p* = 0.0155; (D) Mean number of key presses after the final coin appearance within a round: HE condition, *p* = 0.0827, and LE condition, *p* = 0.0031. The majority revealed significant deviations from normality, necessitating the use of non-parametric testing procedures. For each measure we thus conduct Wilcoxon^25^ signed-rank tests for equality of matched-pairs between effort conditions. In the results that follow, we report the significance level, *p*, and appropriate effect size measure^26,27^, *r*, from each test, and the medians for each condition.

**Figure 1:**
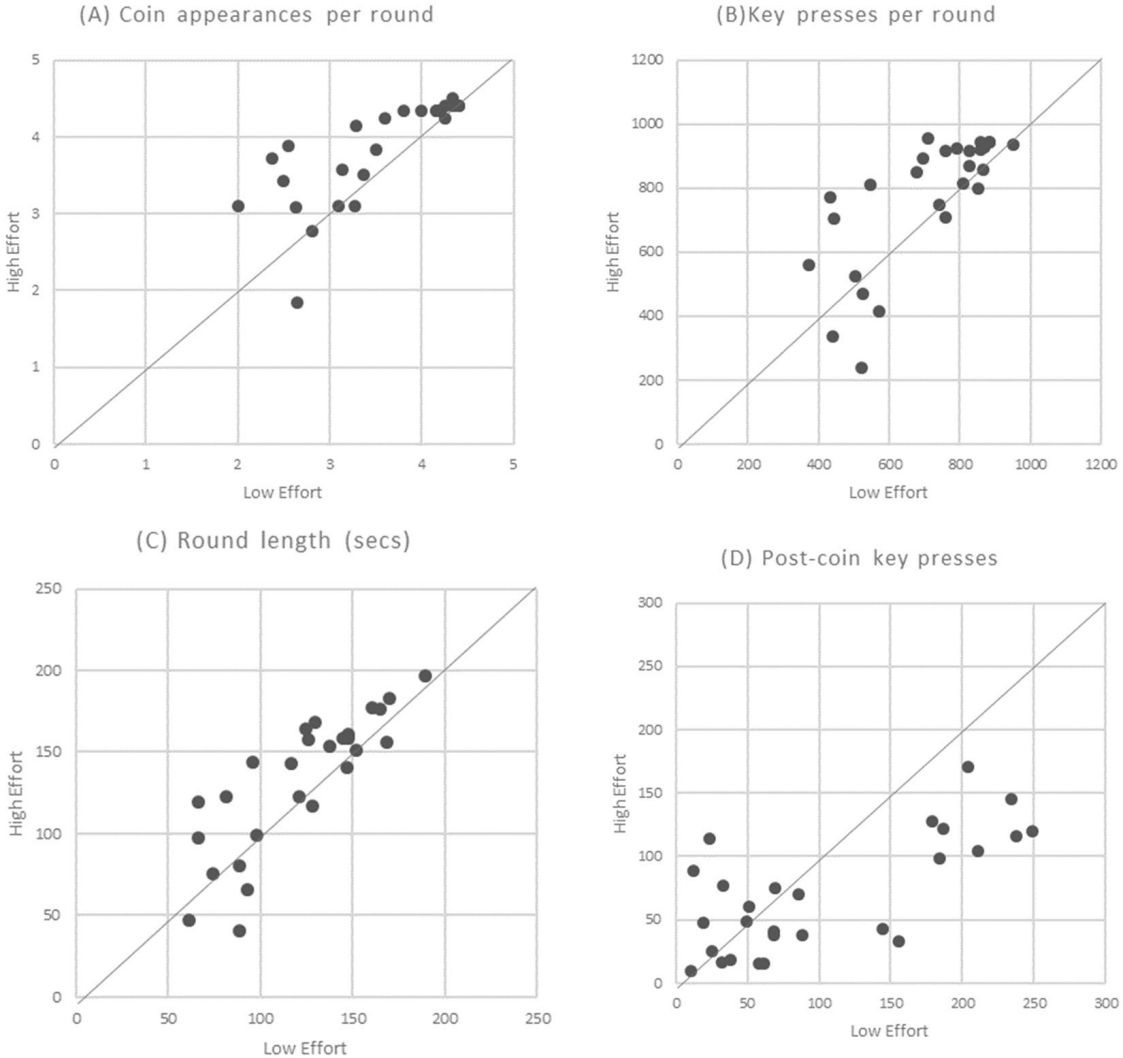
Individual-level data. Each point represents one participant’s set of mean measures for selected variables for each of the two conditions: mean measure for High Effort condition is shown on the Y axis; mean measure for Low Effort condition is shown on the X-axis. A point on the 45° line indicates equality between the two measures; that is, if the mean of each of the participant’s measures did not differ between conditions. The variables shown are calculated separately for each condition and include: (**A**) Mean number of coin appearances per round; (**B**) Mean number of key presses per round; (**C**) Mean round length, in seconds; (**D**) Mean number of key presses after the final coin appearance within a round.

**Figure 2:**
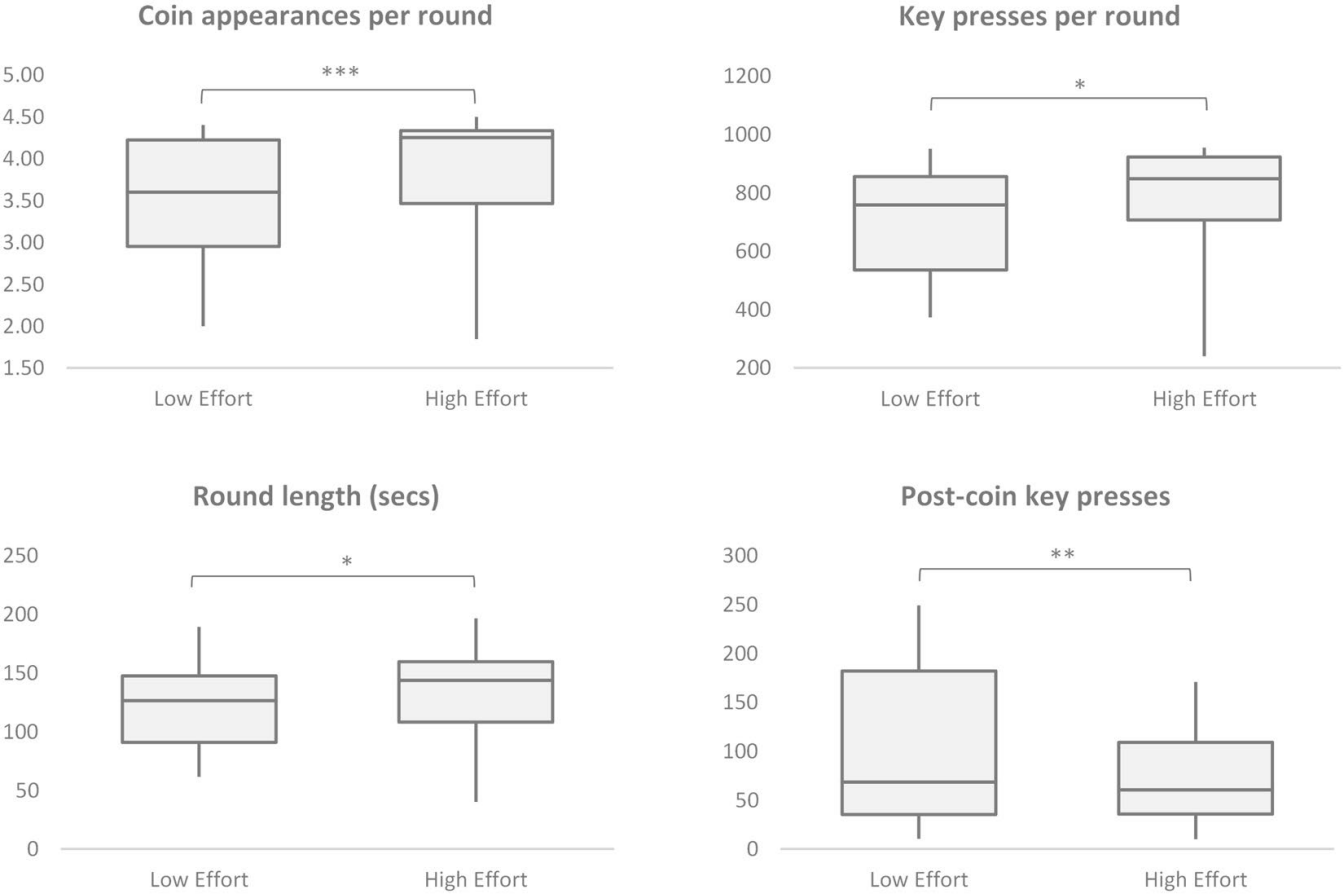
Box plots for effort and performance measures; for High and Low effort conditions. Significance levels from Wilcoxon signed-rank test on matched pairs: ^***^p < 0.001; ^**^p < 0.01; ^*^p < 0.05; ns = non-significant.

Each performance and persistence measure reveals significant differences between conditions (see Figs 1 and 2). The number of coin appearances is significantly higher in the HE condition (*Mdn* = 4.25) than in the LE condition (*Mdn* = 3.60), *p* = 0.0005, *r* = 0.471. Participants persist for a longer time (seconds) in the HE condition (*Mdn* = 143.57) than the LE condition (*Mdn* = 126.33), *p* = 0.0211, *r* = 0.314. And participants move the line further by pressing the key more times in the HE condition (*Mdn* = 847.83) than the LE condition (*Mdn* = 758.60), *p* = 0.0198, *r* = 0.317. All three measures show an increase of between 12–18% when moving from LE to HE condition rounds. Participants also have fewer key presses post the final coin in the round in the HE condition (*Mdn* = 60.65) than LE condition (*Mdn* = 68.50), *p* = 0.0062, *r* = −0.373.

**Figure 3:**
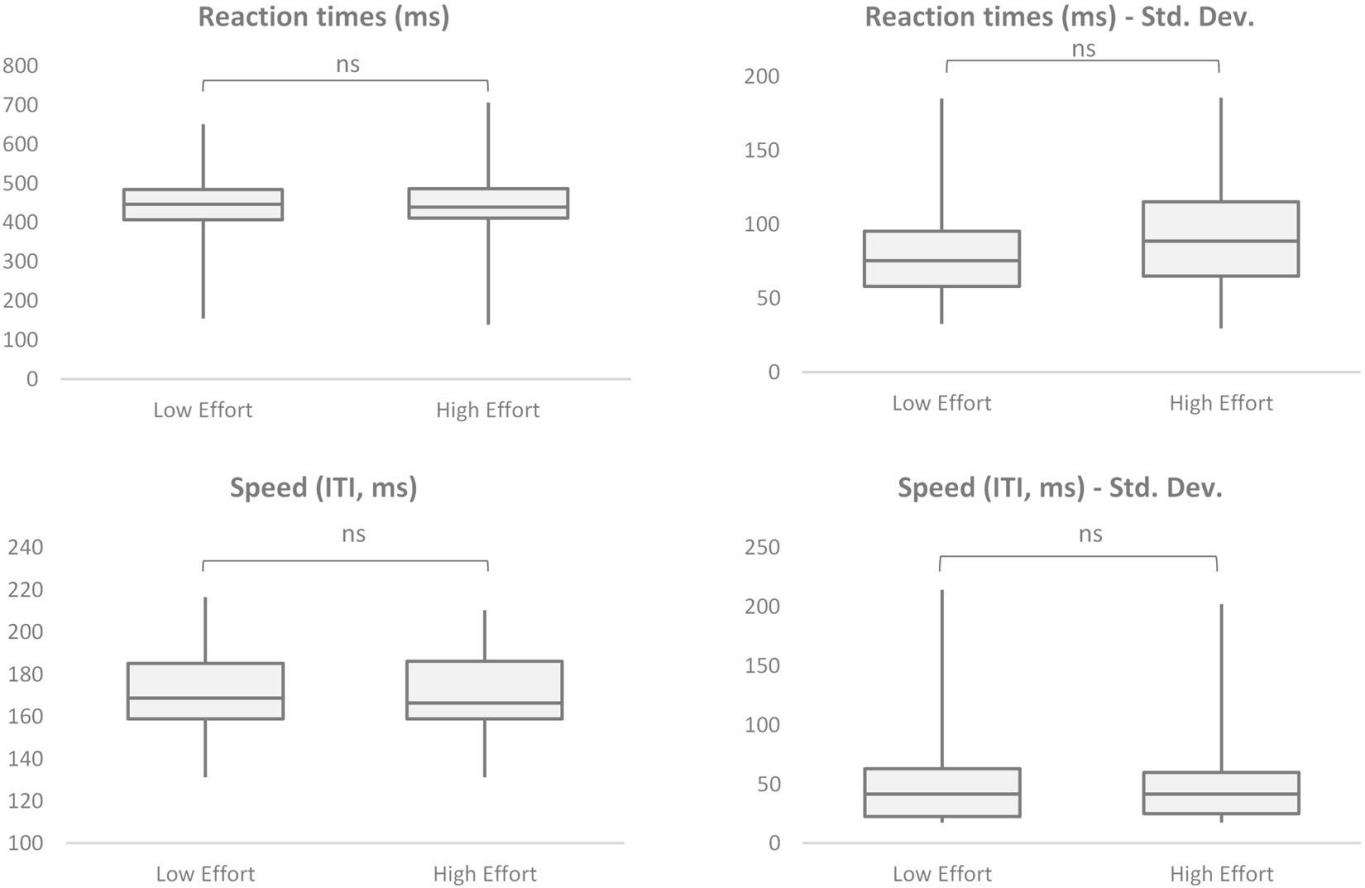
Box plots for executive control measures; for High and Low effort conditions. Significance levels from Wilcoxon signed-rank test on matched pairs: ^***^p < 0.001; ^**^p < 0.01; ^*^p < 0.05; ns = non-significant.

Results from our measures for executive control contrast with those above (see Fig. 3). Reaction times in catching coins are not significantly different in the HE condition (*Mdn* = 439.09) than in the LE condition (*Mdn* = 446.29), *p* = 0.7366, *r* = 0.046. Our comparison of the Standard Deviation in reaction times across conditions show the amount of variation did not systematically differ in the HE condition (*Mdn* = 88.76) from the LE condition (*Mdn* = 75.34), *p* = 0.1128, *r* = 0.216. To analyse key-press speed, we looked at the mean inter-tap interval (ITI). The ITI is the time, in milliseconds, between each press of the key to move the line. A lower average ITI implies quicker speed. The results revealed that key press speed did not significantly differ in the HE condition (*Mdn* = 166.45) than the LE condition (*Mdn* = 168.78), *p* = 0.6480, *r* = −0.062. Further, we find no evidence of change in the consistency of speed between conditions. The Standard Deviation in ITI did not significantly differ between the HE condition (*Mdn* = 41.55) and LE condition (*Mdn* = 41.52), *p* = 0.2391, *r* = −0.160.

## Discussion

We investigated people’s willingness to invest effort in a cooperative activity in which both they and their partner must invest effort to generate rewards to be shared between them. Our prediction was that a higher perceived effort contribution from a partner would cause participants to become more committed to the cooperative activity. Using a within-subject design to control for potential motivation and ability across participants^28^, our results revealed that participants did indeed apply significantly more effort themselves when they perceived their partner to have invested a high degree of effort than when they perceived their partner to have invested a low degree of effort; persisting for longer, moving the line further along and, ultimately, recovering more coins and earning more points. The magnitude of these effects is consistent across the different measures. This provides support for the hypothesis that others’ investment of effort elicits a sense of commitment to cooperative activities.

These findings build upon recent research which has shown that people persist longer in cooperative activities which involve spatio-temporal coordination, waiting for longer periods before ending rounds when they perceive their partner to have invested high relative to low effort^29^. Our results here affirm that perception of effort modulates a sense of commitment. Our findings extend this research in two ways. First, we focus on an activity that lacks strong spatio-temporal coordination. Unlike Székely and Michael (2018)^29^, whose participants worked together with a partner to jointly navigate a snake to collect apples (and earn points), our experiment involved only very low coordination demands. Participants performed separate, though complementary, tasks. Our results therefore put us in a position to infer that the effects of the perception of a partner’s effort upon commitment do not presuppose a context involving a high degree of coordination. Second, our task requires participants to continuously apply effort rather than just patiently wait. While previously waiting time was used to operationalise commitment, our design requires participants not only to wait but to pay an effort cost. Our findings therefore indicate that the sense of commitment elicited by the perception of a partner’s effort is sufficient to increase people’s willingness to invest time and effort in a cooperative activity.

Our study was driven by a valuation perspective of effort-motivated cognitive control^28,30–32^. Participants generate subjective expectations of the benefit associated with their task – the expectation of points associated with token appearances – but must pay an effort cost in order obtain that benefit. Crucially, this effort cost increases as the round progresses: Each subsequent key press moves the line forward by a decreasing amount, with the effort cost of continuing the round thus increasing as the line progresses. Participants thus face an increasing cost function, while the subjective valuation of the task remains constant as they are all primed with the same benefit expectation; they believe that tokens appear at random intervals, regardless of the round number or time spent in the round. Continuous monitoring processes that allow individuals to see if current actions are having a desired effect form one minimum requirement in making joint-action possible^33^. Participants must have sufficient feedback on their decisions to influence their resource budgets and allocation for a task^34^, as in our experiment, where subjects receive instantaneous feedback on their effortful actions through the line movement and coin reaction outcome. When the marginal cost of continuing in the round is greater than the expected marginal benefit of coin appearance, participants will thus exit the round^32^. Our experiment exploited this behaviour: given that the cost structure remains constant across both conditions, differences in effort provision in HE and LE rounds must be driven by a change in benefit valuation within rounds. Our results are consistent with the hypothesis that a sense of commitment is the driver of this change: participants’ value function is systematically greater in HE than LE rounds.

Our results show no significant difference between HE and LE rounds in reaction times to coin appearances or in the speed at which participants moved the line. We similarly find no difference across conditions in the within-round variation of these measures. This may indicate that these measures were not sufficiently sensitive to pick up any differences in the extent to which executive control was engaged between our two conditions. And indeed, this explanation is consistent with the small number of coins missed: only 54 (<1%) out of a total 5,412 coin appearances across all subjects were not caught – this does not differ greatly between HE (*n* = 30) and LE (*n* = 24) conditions. Similarly, a further reason why we may not have observed any significant differences in our speed and reaction time measures is that increased persistence in the HE rounds may been traded off with other effort-requiring actions, such as slower speed and reaction times.

It therefore remains an open question for future research to investigate the influence of a sense of commitment on executive function. One possibility would be to implement a more attention-demanding task to tease apart the influence of cognitive control and subjective valuation drivers of behaviour related to increased commitment. A further important challenge would be to investigate the neural underpinnings of the motivational effects of the sense of commitment to another. In this regard, previous research provides empirical evidence that the temporal parietal junction may play a central role in mediating individuals’ willingness to invest effort in collaborative activity^35^.

It would also be interesting for future research to probe perceptions of relative effort contribution in cooperative activities. While we are generally willing to exert effort for others’ benefit^22^, the extent of our generosity may be mediated by our perception of relative resource commitments. For example, we are likely to be less willing to contribute earned income to individuals or groups when we perceive our own effort contribution to be greater than others’^36,37^. To prevent participants from being able to directly match their partner’s level of effort, our experiment involved different tasks with different kinds of effort. While our within-subject design deals with unobserved factors related to participants’ own views of the relative effort required in each task (participants may believe their task to be more difficult than their partners’), it would be interesting to directly investigate the effects of perceived fairness of effort distribution on the sense of commitment, and also to explore the effects on commitment of different types of perceived partner effort contribution, such as physical effort.

## Methods

### Participants

Using G*Power 3.1^38^, a sample size of 27 was calculated to provide 80% statistical power for detecting a medium-sized effect equivalent to what we observed in a pilot study (*d* = 0.58), assuming a two-tailed t-test and an alpha level of 0.05. The experiment was conducted in groups of 4 at a time, with a total of 28 participants taking part in the experiment (the 28th participant to sign up chronologically was excluded prior the analyses from the sample to reach the optimal sample size). In addition to the 27 participants who constitute our sample (15 females; Age: M = 21.41, S.D. = 2.10), thirteen further participants did not meet threshold participation requirements and were excluded prior to analysis. All participants were recruited through the University of Warwick SONA System, a voluntary sign-up system available to anyone interested in taking part in paid research conducted by Warwick researchers. Exclusion criteria included participation in previous experiments within the same umbrella project. All participants reported speaking and understanding English.

Each participant was provided with an information sheet outlining the overall project of which the experiment was part as well as instructions for the experiment. Participants also provided written informed consent prior to the experiment. Ethics clearance for the experiment was obtained from the University of Warwick’s Humanities and Social Sciences Research Ethics Committee (HSSREC) and all methods were performed in accordance with the relevant guidelines and regulations. In addition to the instructions, participants were told that they would be paired with a partner, the total points accumulated by their pair shared equally between them at the end. Participants were instructed that both their own and their partner’s monetary payments at the end of the experiment would depend on the points accumulated during their task, which would vary between £6 and £12.

### Apparatus and Stimuli

Stimulus presentation was programmed on a PC using Cycling ‘74 Max Live version 7.3.4. Participants input into the task was captured using a standard QWERTY keyboard and the computer screen provided participants with real-time visual feedback on their inputs. An instruction sheet provided showed the meaning of relevant keys. During rounds, on the same screen, participants saw a video of what they perceived as their partner completing her task. The video was designed to behave in a human-like manner, with delays and mistakes included in completing the task. To control for learning effects, half the participants experienced one chronology of rounds and the other half a mirror version.

### Procedure

Participants were first seated together in groups of four, and instructions were read aloud by the experimenter. The group was informed that each person would be privately, randomly assigned either the role of Player A or Player B, and that each would be partnered with another in the group taking the opposite role, although they would not know exactly which of the remaining three participants this would be. Participants were advised to read the instructions for both roles (Player A and Player B). Each participant was then shown to a separate adjoining cubicle, containing information on their role allocation (all were allocated the role of Player A) and a PC on which they would complete their task.

Participants – all in the role of Player A – had 30 minutes to complete as many rounds as they wished, with the instructed goal of earning as many points as possible. Their partner – Player B – would perform a different task to ‘unlock’ the next round of Player A’s task. The role of Player B was pre-programmed. Points earned by Player A would be automatically shared equally by both players. Time was paused in between rounds while Player B completed her task and participants were informed that Player B would not be able to watch her performance during her task.

**Figure 4:**
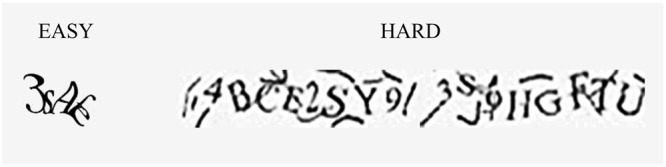
Sample captchas. These two examples were presented to participants in the instructions prior to beginning the experiment.

Player B’s task was ostensibly to decipher a captcha key she had been given to unlock the next round for Player A (see Fig. 4). A new captcha key was required to be deciphered at the beginning of each round and Player A had to wait until the task was complete before proceeding to the next stage and getting the opportunity to earn more points. Player A was shown two outputs on her screen. First, Player B’s rating of the level of difficulty of the captcha to be deciphered. Captchas ratings were presented to Player A on a visual scale, with the following possibilities: very easy; easy; medium; hard; very hard. Second, asterisks denoting Player B’s “work-in-progress” followed by the completed key once it was unlocked.

**Figure 5:**
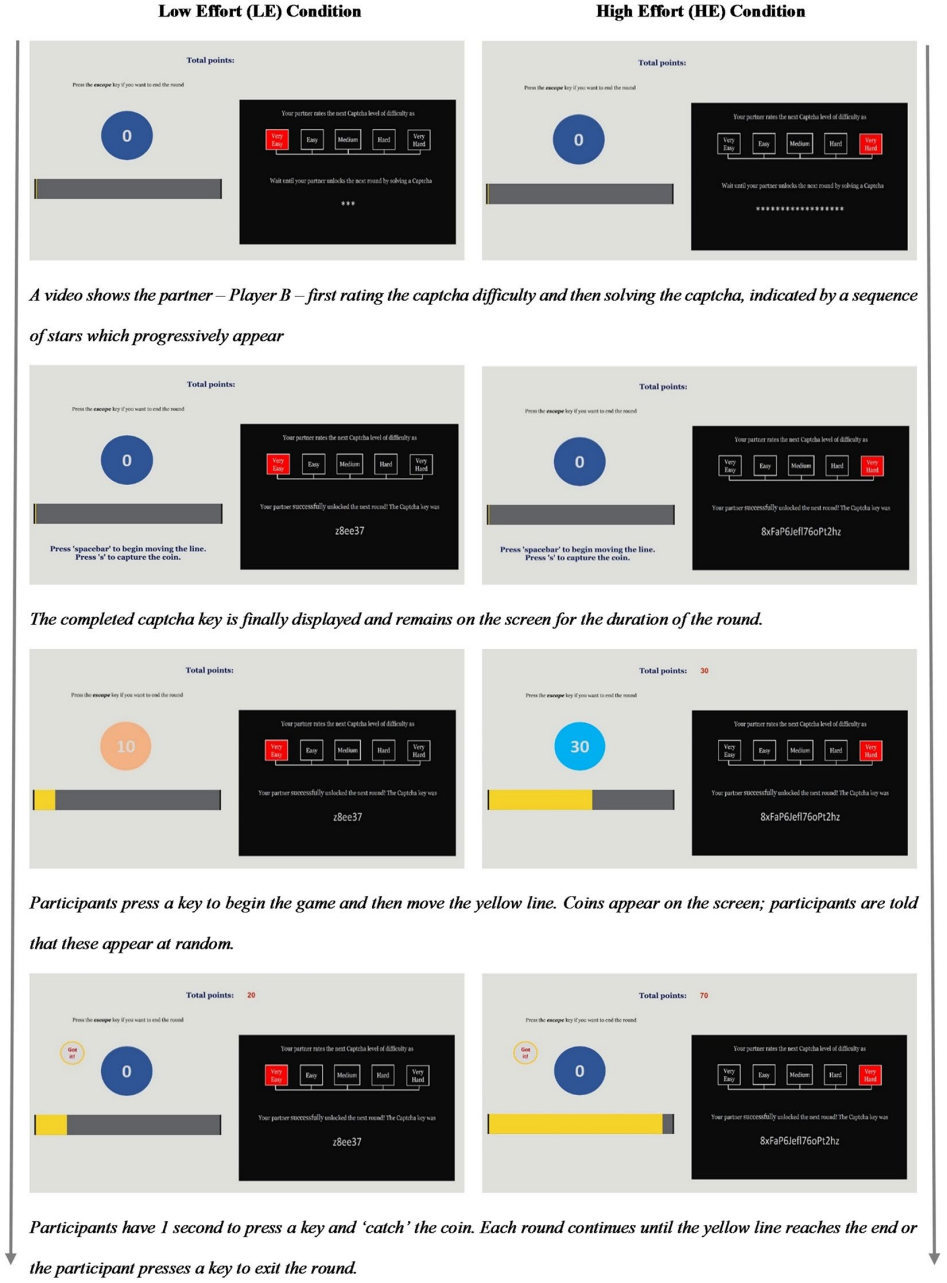
Trial structure. Each trial consisted of a captcha phase (Player B’s task to ‘unlock’ the round), followed by a round of the coin catching game (Player A’s task to get points for the team).

Once unlocked, Player A could begin her round (see Fig. 5). Participants applied effort to the task by continuously pressing the *‘spacebar’* key. This moved a yellow line on the screen. To make the task increasingly boring and/or more difficult, the line got progressively harder to move the further it was along (requiring more presses per unit of distance). On the screen, a coin worth 0 points would periodically change into a new coin starting at 10 and increasing in multiples of 10 to 60 points. Whether and when a new coin appeared was said to be randomly determined, although patterns of appearance were pre-built into the program. Participants could therefore never be certain if and when a new coin would appear. When a new coin appeared, participants were given one second to press the *‘s’* key to catch, or sell, the coin as quickly as possible before it disappeared. Successfully catching the coin would add its points to a running total; missing the coin resulted in no points being added. Participants – in the role of Player A – had one practice round with no time limit imposed and in which points did not count towards the final tally. Monetary payment was thus allocated based on the number of points earned by participants (as Player A). The appearance of coins varied independently across rounds and was not determined by participant performance. Participants were given the option of exiting the current round before the line had reached the end by pressing the *‘escape’* key. This would enable them to begin a new round, but only after it had been ‘unlocked’ by their partner (Player B) in performing her task.

## Data Availability

The datasets generated during and/or analysed during the current study are available in the *Effortinvest_2018* repository, available online at http://dx.doi.org/10.17605/OSF.IO/VGQ3S.

## References

[1] Drias, H., Sadeg, S. & Yahi, S. Cooperative bees swarm for solving the maximum weighted satisfiability problem. *Computational Intelligence and Bioinspired Systems*, 417–448 (2005).

[2] Seed M, Clayton NS, Emery NJ (2008). Cooperative problem solving in rooks (corvus frugilegus). Proceedings of the Royanl Society of London B: Biological Sciences.

[3] Melis AP, Hare B, Tomasello M (2006). Chimpanzees recruit the best collaborators. Science.

[4] Tomasello, M. *Why we cooperate*. (MIT Press, 2009).

[5] Melis AP, Semmann D (2010). How is human cooperation different?. Philosophical Transactions of the Royal Society (Biological Sciences)..

[6] Sterelny, K. *The evolved apprentice*. (MIT Press, 2012).

[7] Henrich, N. & Henrich, J. *Why humans cooperate: a cultural and evolutionary explanation*. (Oxford Univeristy Press, 2007).

[8] Nowak MA (2012). Why we help. Scientific American.

[9] West SA, Griffin AS, Gardner A (2007). Social semantics: altruism, cooperation, mutualism, strong reciprocity, and group selection. Journal of Evolutionary Biology.

[10] Kurzban R, Burton-Chellew MN, West SA (2015). The evolution of altruism in humans. Annual Review of Psychology.

[11] Hamilton WD (1963). The evolution of altruistic behaviour. The American Naturalist.

[12] Maynard-Smith J (1964). Group selection and kin selection. Nature.

[13] Trivers RL (1971). The evolution of reciprocal altruism. The quarterly review of biology.

[14] Nowak MA, Sigmund K (1998). Evolution of indirect reciprocity by image scoring. Nature.

[15] Roberts G (2005). Cooperation through interdependence. Animal Behaviour.

[16] Boyd, R. & Richerson, P. J. In *Better than Conscious? Decision Making*, *the Human Mind*, *and Implications for Institutions*. 305–324 (MIT Press, 2008).

[17] Fehr E., Schmidt K. M. (1999). A Theory of Fairness, Competition, and Cooperation. The Quarterly Journal of Economics.

[18] Battigalli P, Dufwenberg M (2007). Guilt in Games. The American Economic Review.

[19] Heintz C, Celse J, Giardini F, Max S (2015). Facing expectations: those that we prefer to fulfil and those we disregard. Judgement and Decision Making.

[20] Braun Daniel A., Ortega Pedro A., Wolpert Daniel M. (2009). Nash Equilibria in Multi-Agent Motor Interactions. PLoS Computational Biology.

[21] Braun DA, Ortega PA, Wolpert DM (2011). Motor coordination: when two have to act as one. Experimental brain research.

[22] Lockwood P, Husain M, Apps M (2017). Prosocial apathy for helping others when effort is required. Nature: Human Behaviour.

[23] Michael J, Sebanz N, Knoblich G (2016). The sense of commitment: a minimal approach. Frontiers in Psychology.

[24] Miyake A, Friedman N, Emerson M, Witzki A, Howerter A (2000). The unity and diversity of executive functions and their contributions to complex “frontal lobe” tasks: a latent variable analysis. Cognitive Psychology.

[25] Wilcoxon F (1945). Individual comparisons by ranking methods. Biometrics.

[26] Field, A., Miles, J. & Field, Z. *Discovering statistics using R*. (Sage, 2012).

[27] Rosenthal, R. *Meta-analytic procedures for social research (2nd ed*.*)*. (Sage, 1991).

[28] Botvinick M, Braver T (2015). Motivation and Cognitive Control: From Behavior to Neural Mechanism. Annual Review of Psychology.

[29] Székely M, Michael J (2018). Investing in commitment: Persistence in a joint action is enhanced be the perception of a partner’s effort. Cognition.

[30] McGuire JT, Kable JW (2015). Medial prefrontal cortical activity reflects dynamic re-evaluation during voluntary persistence. Natural Neuroscience.

[31] Apps, M. A. J., Grima, L. L., Manohar, S. & Husain, M. The role of cognitive effort in subjective reward devaluation and risky decision-making. *Scientific Reports*, **5** (2015). 10.1038/srep16880 PMC4653618 26586084

[32] DellaVigna, S. & Pope, D. What motivates effort? Evidence and expert forecasts. *Review of Economic Studies*, *rdx*, **033** (2017).

[33] Vesper, C., Butterfill, S., Knoblich, G. & Sebanz, N. A minimal architecture for joint action. *Neural Networks*, (2010). 10.1016/j.neunet.2010.06.002 20598504

[34] Thaler R (1999). Mental Accounting Matters. Journal of Behavioural Decision Making.

[35] Le Bouc R, Pessiglione M (2013). Imaging social motivation: distinct brain mechanisms drive effort production during collaboration versus competition. The Journal of Neuroscience.

[36] Erkal N, Gangadharan L, Nikiforakis N (2011). Relative earnings and giving in a real-effort experiment. American Economic Review.

[37] Cherry TL, Frykbom P, Shogren JF (2002). Hardnose the dictator. American Economic Review.

[38] Faul F, Erdfelder D, Buchner A, Lang A-G (2009). Statistical power analysis using G*Power 3.1: tests for correlation and regression analysis. Behaviour Research Methods.

